# Perioperative Antithrombotic Strategies in Vascular Surgery: A Survey in Germany

**DOI:** 10.1002/hsr2.70732

**Published:** 2025-04-18

**Authors:** Dmitriy I. Dovzhanskiy, Moritz S. Bischoff, Karola Passek, Hinrich Böhner, Dittmar Böckler

**Affiliations:** ^1^ Department of Vascular and Endovascular Surgery University Hospital Heidelberg Heidelberg Germany; ^2^ St. Rochus Hospital Castrop‐Rauxel, Katholisches Krankenhaus Dortmund‐West Castrop‐Rauxel Germany

**Keywords:** anticoagulation, atithrombotics, perioperative management, risk stratification, survey, vascular surgery

## Abstract

**Background and Aims:**

The variety of modern antithrombotic medications complicates the choice of individual perioperative therapy in vascular surgery, especially when more than one antithrombotic option is possible. The aim of this study was to determine the perioperative and periinterventional setting concerning antithrombotics in vascular surgery in Germany.

**Methods:**

This article is based on a survey from year 2018 of heads of German vascular surgical departments or units regarding their experience with different anticoagulants. The survey asked for the frequency and time of preoperative pausing of the antithrombotics before various vascular operations or interventions.

**Results:**

The evaluable response rate was 52% (169/324). Acetylsalicylic acid was discontinued before open aortic surgery in 9% (15/169) of respondents. Clopidogrel was paused in 65% (107/169) before open aortic surgery, in 25% (41/169) before vascular surgery (like carotid endarterectomy, endovascular aortic repair, or operations on peripheral arteries), and in 11% (18/169) before peripheral percutaneous interventions. Discontinuation of vitamin K antagonists or direct oral anticoagulants (took place before conventional operations in 99.4%; oral anticoagulation was continued for peripheral percutaneous interventions in only 6% (8/169). Management was heterogeneous with regard to the timing of the perioperative medication pause. Clopidogrel was not discontinued according to time specifications in 8%; ticagrelor in 75%; rivaroxaban in 23%; and dabigatran in 29%, compared to the recommendations of the industrial information sheets.

**Conclusion:**

The perioperative antithrombotic therapy in German vascular surgery clinics is not uniform and does not correspond to the current specialist recommendations in a notable proportion of clinics.

## 1 Introduction

The variety of modern anticoagulant and antiplatelet medications [1] makes the choice of appropriate therapy more complicated, especially in invasive medical fields including vascular surgery. Vascular surgery patients in particular are usually taking several antithrombotic drugs due to their cardiological comorbidities and the number of patients with indication for combination antiplatelet and oral anticoagulant therapy is increasing [2].

A short time ago, the usual preparations included acetylsalicylic acid (ASA), clopidogrel, phenprocoumon, and heparin. Today, a wealth of new anticoagulants and platelet aggregation inhibitors have been introduced with variable combination options. The recently published clinical practice guideline finally give evidenced based recommendations including for perioperative setting [1] after a long time of off‐label use of antithrombotics.

Based on the daily doses prescribed via statutory health insurance, at least one million patients in Germany received oral anticoagulation (OAC) and at least 900,000 patients received therapy with a platelet aggregation inhibitor in 2022. Of the prescribed OAC daily doses, 88% are direct oral anticoagulants (DOACs: apixaban, dabigatran, edoxaban, and rivaroxaban), with a proportion of patients taking vitamin K antagonists (VKA, in Germany mainly phenprocoumon) [3].

Bleeding complications are the most frequent and relevant side effects of OAC or platelet aggregation inhibitor use [1, 4]. Thus, for every indication where antithrombotic therapy is recommended, the harm caused by potential major bleeding must be considered [1].

With regard to patients who take antithrombotics and are about to undergo vascular surgery, the treating physicians usually see the following three basic scenarios: (1) elective vascular surgery with low risk of bleeding (e.g., peripheral interventions, vascular access surgery); (2) elective vascular surgery with a high risk of bleeding (e.g., bypass operation, carotid or aortic surgery); or (3) urgent or emergency vascular surgery associated with an increased risk of procedural bleeding (e.g., critical limb ischemia). Despite there are many risk prediction scores for assessing an individual's bleeding risk, they are not well validated or widely used in the vascular surgery patient population [1]. So, till now, vascular surgeons can only individual estimate the perioperative risk of bleeding versus thromboembolic complications.

The aim of this study was to determine the perioperative or periinterventional antithrombotic therapy in vascular surgery in Germany.

## 2 Materials and Methods

In 2018, a questionnaire was sent by post to 324 identified vascular surgery departments or units in Germany. These were identified by mean of internal database and the website www.kliniken.de. The questionary was an independent part of the project to the perioperative care in vascular surgery of the same working group; the results concerning preoperative cardiac evaluation were published before [5].

The survey consisted of two main sections: (1) the frequency of, and (2) the timing of the preoperative pause of anticoagulants or antiplatelet drugs.

In the first section, the questionnaire asked whether ASA, Clopidogrel, VKA, or DOACs were routinely stopped before (a) open abdominal aortic surgery (OAR), (b) other vascular surgeries (VS) such as carotid endarterectomy, endovascular aortic repair (EVAR), or operations on peripheral arteries, and (c) peripheral percutaneous interventions for lower extremity peripheral arterial disease.

In the second section, the questionnaire asked how many days before surgery the respondent would pause ASA, clopidogrel, ticagrelor, VKA, rivaroxaban, or dabigatran before (a) OAR and (b) VS.

Additionally, the questionnaire asked about the characteristics of the participating clinics: the organizational structure of the vascular surgery and affiliation to the German hospital service level (basic and standard care hospital, maximum care hospital, central care hospital, or university hospital).

As a survey of vascular surgeons and not the study on patients, the ethical approvement was not required.

Microsoft Office Excel 2013 (Microsoft, Redmond, USA) was used for analysis. Results are presented descriptively.

## 3 Results

### 3.1 Characteristics of the Participants

The response rate was 52% (169/324). In 20 responses there were some missing answers, concerning timing of the preoperative drugs pause.

Most of the participating respondents stated that they belonged to a central service hospital (41%, 68/169); 28% (48/169) belonged to a basic and standard care hospital; 22% (377169) to a maximum care hospital; and 9% (16/169) to a university hospital (Table 1).

**Table 1:** Characteristics of the participants.

Hospital service level	*n* = 169
University hospital	16 (9%)
Maximum care hospital	37 (22%)
Central care hospital	68 (41%)
Basic and standard care hospital	48 (28%)

140 (83%) respondents represented independent departments of vascular surgery, and the remaining respondents were parts of general or cardiac surgery units.

### 3.2 The Frequency of Preoperative Pause of Antithrombotics

Before OAR, ASA was paused in 15 (9%), clopidogrel in 107 (65%), VKA in 168 (99.4%), and DOACs in 167 (98.8%) answers (Table 2).

**Table 2:** The frequency of preoperative pause of anticoagulant or antiplatelet drugs before conventional abdominal aortic surgery (*n* = 169 respondents).

Drug	Paused	Continued
ASA	15 (9%)	154 (91%)
Clopidogrel	107 (65%)	57 (35%)
Vitamin K antagonists	168 (99.4%)	1 (0.6%)
DOACs	167 (98.8%)	2 (1.2%)

Abbreviations: ASA, acetylsalicylic acid; DOACs, direct oral anticoagulants.

Before VS, ASA was not paused in any of the answers. Clopidogrel was stopped in 41 (25%, where four respondents would do this only in the case of dual antiplatelet therapy [DAP]); VKAs in 162 (96%); and DOACs in 164 (98%) respondents (Table 3).

**Table 3:** The frequency of preoperative pause of anticoagulant or antiplatelet drugs before other vascular operations like carotid endarterectomy, EVAR, peripheral artery surgery.

Drug	Paused	Continued
ASA	0	169 (100%)
Clopidogrel	41 (25%)	124 (75%)
Vitamin K antagonists	162 (96%)	6 (4%)
DOACs	164 (98%)	3 (2%)

Abbreviations: ASA, acetylsalicylic acid; DOACs, direct oral anticoagulants; EVAR, endovascular aortic repair.

Before percutaneous interventions, ASA was not stopped in any of the answers. Clopidogrel was paused in 18 (11%), where 4 respondents would do this only in the case of DAP therapy, VKAs in 162 (96%) and DOACs in 159 (94%) respondents (Table 4).

**Table 4:** The frequency of preoperative pause of anticoagulant or antiplatelet drugs before percutaneous interventions.

Drug	Paused	Continued
ASA	0	169 (100%)
Clopidogrel	18 (11%)	150 (89%)
Vitamin K antagonists	162 (96%)	6 (4%)
DOACs	159 (94%)	8 (6%)

Abbreviations: ASA, acetylsalicylic acid; DOACs, direct oral anticoagulants.

### 3.3 Timing of Preoperative Antithrombotic Drugs Pause

The timings of the preoperative pause of antithrombotics are shown in Table 5.

**Table 5:** The timing of preoperative pauses of anticoagulant or antiplatelet drugs.

	Day before OP	Conventional abdominal aortic surgery	Other vascular operations such as carotid endarterectomy, EVAR, or peripheral artery surgery
ASA	Continued	140 (84%)	154 (92.8%)
	Day 1–4	4 (2.6%)	2 (1.2%)
	Day 5	9 (5.4%)	4 (2.4%)
	Day 7	9 (5.4%)	5 (3.0%)
	Day 10	4 (2.6%)	1 (0.6%)
	**Evaluable answers**	***n*** = **166**	***n*** = **166**
Clopidogrel	Continued	48 (29%)	91 (56%)
	Day 1–4	14 (8%)	9 (5%)
	Day 5	36 (22%)	25(15%)
	Day 7	50 (30%)	30 (18%)
	Day 8–10	17 (11%)	10 (6%)
	**Evaluable answers**	***n*** = **165**	***n*** = **165**
Ticagrelor	Continued	9 (6%)	21 (14%)
	Day 1–2	30 (20%)	28 (19%)
	Day 3–5	51 (34%)	49 (33%)
	Day 7	49 (33%)	41 (28%)
	Day 8–10	11 (7%)	9 (6%)
	**Evaluable answers**	***n*** = **150**	*n* = 148
Rivaroxaban	Continued	0 (0%)	4 (2%)
	Day 1	34 (20%)	37 (23%)
	Day 2	83 (50%)	78 (47%)
	Day 3	19 (13%)	18 (11%)
	Day 4–5	14 (8%)	13 (8%)
	Day 7–10	4 (2%)	4 (2%)
	Adapted to renal function	11 (7%)	11 (7%)
	**Evaluable answers**	***n*** = **165**	***n*** = **165**
Dabigatran	Continued	0 (0%)	3 (2%)
	Day 1	28 (17%)	32 (20%)
	Day 2	77 (47%)	73 (45%)
	Day 3	26 (16%)	25 (15%)
	Day 4–5	16 (10%)	13 (8%)
	Day 7–10	5 (3%)	6 (4%)
	Adapted to renal function	10 (7%)	10 (6%)
	**Evaluable answers**	***n*** = **162**	***n*** = **162**
Vitamin K antagonists	Continued	0 (0%)	2 (1%)
	Day 1–2	8 (5%)	6 (3%)
	Day 3–4	29 (18%)	32 (20%)
	Day 5–6	53 (32%)	52 (32%)
	Day 7–10	62 (37%)	60 (36%)
	Adjusted to INR	14 (8%)	13 (8%)
	**Evaluable answers**	***n*** = **166**	*N* = 165

Abbreviations: ASA, acetylsalicylic acid; DOACs, direct oral anticoagulants; EVAR, endovascular aortic repair.

ASA was mostly not paused before OAR (84%) or VS (92.8%). The remaining respondents (16% and 7.2%, respectively) paused the medications between days 1 and 10 preoperatively.

Clopidogrel was not paused in around one‐third (29%) before OAR and in about half before VS (56%). In contrast, clopidogrel was paused on day 5 (22% OAR, 15% VS) and on Day 7 (30% OAR, 18% VS) in around half of departments/units.

Few respondents continued ticagrelor treatment when performing elective vascular surgery (6% OAR, 14% VS). About one‐third of respondents would pause the medication for 3–5 days preoperatively (34% OAR, 33% VS) and another third for 7 days (OAR 33%, VS 28%).

None of the included departments/units would perform OAR, and only 2% VS, with ongoing rivaroxaban treatment. Most respondents would pause the drug 2 days (OAR 50%, VS 47%) or 1 day (OAR 20%, VS 23%) before surgery. Responses regarding dabigatran were very similar. None of the departments/units would perform OAR, and only 2% would perform VS, without pausing this medication. About half of the respondents paused dabigatran 2 days before surgery (OAR 47%, VS 45%), and a smaller proportion for 1 day before surgery (OAR 17%, VS 20%). Similar to rivaroxaban, 7% of respondents stated that the decision was based on renal function.

VKAs were paused in all departments/units before OAR and in 99% s before versus About one‐third of respondents paused it 5–6 days (both OAR and VS 32%) or 7–10 days (OAR 37%, VS 36%) before surgery.

## 4 Discussion

For every indication where antithrombotic therapy is recommended, the benefit of it use must be in balance with potential harm, mainly via major bleeding events [1]. In the recent study we determine the management of antithrombotic drugs before elective vascular surgery in Germany by survey. Because of the large number of new drugs, we could not evaluate the use of all registered used antiplatelet and anticoagulant medications. We found that the management of these drugs to the timepoint of the survey was heterogeneous and often does not correspond to the current specialist recommendations in a notable proportion of clinics.

ASA is a common drug well known to patients and physicians for the treatment of symptomatic vascular disease [6]. In established atherosclerotic disease, ASA is associated with significant reductions in the rate of serious vascular events, including stroke and coronary events, and a 10% reduction in total mortality [7]. Established monotherapy with ASA or thienopyridines (e.g., clopidogrel) is recommended to be continued during the peri‐operative period after open or endovascular abdominal aortic aneurysm (AAA) repair [8]. Moreover, antiplatelet monotherapy with ASA or thienopyridines (e.g. clopidogrel) does not pose an excessive risk of bleeding during AAA repair [9], but ASA non‐adherence/withdrawal has been associated with a three‐fold higher risk of major adverse cardiac events [10]. Nevertheless, contrary to these recommendations, treatment with ASA was reported to be interrupted for elective surgery in around one in ten respondent departments/units.

One‐third of our respondents reported performing OAR, and three quarters of them VS, while continuing clopidogrel treatment. More challenging is the decision to interrupt dual antiplatelet agents. Most current recommendations are based on expert opinion. One of the predictors of major adverse cardiac events appears to be the time elapsed between coronary stent implantation and surgery [11, 12]. The guidelines of the European Society for Vascular Surgery (ESVS) [8] state that in patients taking dual antiplatelet therapy after interventional coronary revascularisation, delaying abdominal aortic aneurysm repair until reduction of dual therapy to monotherapy may be considered. Alternatively, if AAA repair becomes necessary, EVAR may be considered under dual antiplatelet therapy [8]. A systematic review of 11 guidelines found that elective noncardiac surgery should be delayed for at least 4 weeks after bare‐metal stent implantation and at least 6 months after drug‐eluting stent implantation. For elective surgery, all guidelines advised continuing acetylsalicylic acid (ASA) therapy whenever possible [13].

Where interruption of antiplatelet therapy is required, four guidelines advised discontinuation of ASA/clopidogrel at least 5 days before surgery, while two guidelines advised discontinuation 7 to 10 days before surgery. Five guidelines provided variable guidance for the use of perioperative bridging during antiplatelet therapy interruption [13].

Only a small number of departments/units reported performing elective surgery with ongoing ticagrelor treatment. This was the case for VS more often than OAR. Monotherapy with ticagrelor was associated with a lower incidence of bleeding without an increased risk of ischemic events [14], but isn't referred to in the recent recommendations [15]. Nevertheless, ticagrelor is an established dual antiplatelet therapy option [16]. Experience of dual therapy involving more potent antiplatelet agents, such as prasugrel and ticagrelor, and AAA repair is very limited but is likely associated with a high risk of serious bleeding and should be avoided [8]. If required, ticagrelor can be interrupted 2–3 days before surgery without an increased rate of severe bleeding [17].

Both VKAs and DOACS were reported to be paused before elective surgery in practically all responders. However, the time point of therapy interruption is in practice a topic of discussion.

The ESVS guidelines advice that warfarin and new oral anticoagulants should be discontinued at least 5 days and 2 days, respectively, before aortic surgery to mitigate the risk of excessive bleeding. Depending on the indications for use, anticoagulation may be bridged during the peri‐operative period using a short acting agent such as low molecular weight heparin or unfractionated heparin [8]. Generally, the individual approach to DOAC interruption is based on the procedure‐related bleed risk, DOAC type, and patient renal function. According to the pharmacokinetics, they should be stopped 2 days before a high bleeding risk vascular surgery in patients with normal renal function. In patients taking dabigatran with a creatinine clearance < 50 mL/min, the dabigatran should be stopped 4 days before surgery [18]. Remarkably, 2% of respondents did not interrupt DOACs in the case of versus

Two important studies that have been published during or after conceiving of our survey ‐ Compass Trial and VOYAGER Trial [19, 20] ‐ are supporting the combination with ASS 100 mg und low dose Rivoroxaban 2,5 mg in patients with PAD. We could not find any reports about the dealing with this regime in perioperative term. Nevertheless, it would be an interesting question if this combination, if not interrupted, would lead to more perioperative bleeding complications.

In general, the risk of thromboembolic complications should first be evaluated in patients receiving OAC before discontinuing the medication [4]. For example, oral anticoagulation can be temporarily paused with relatively little risk for patients who have had a thrombosis a long time ago (> 3 months) or with atrial fibrillation without concomitant disease [4]. On the other hand, a significant drop in the INR value over several days in patients with a mechanical heart valve or a recent pulmonary embolism is unacceptable due to the risk of fatal events [4, 21, 22]. Patients with a high thromboembolic risk (atrial fibrillation due to valve defects and/or valve replacement; atrial fibrillation with a CHADS2 score ≥ 5; patients with a mechanical heart valve; or history of thromboembolic events within the last 3 months) are usually bridged with heparin [4, 21]. In this case, the VKA should be discontinued 5 days before the planned intervention and, depending on the INR value, anticoagulation with a low molecular weight heparin (LMWH) should be given for 3 days before the intervention [4]. This should then also be paused 24 h before the procedure. Most guidelines do not recommend unfractionated heparin over LMWH [4, 22, 23]: simpler handling, the possibility of outpatient therapy, and the lower risk of bleeding are factors in favor of intermittent subcutaneous injection with LMWH [4].

In patients without a high risk of thromboembolic complications (non‐valvular atrial fibrillation, CHADS2 score < 5, previous thrombosis > 3 months), phenoprocoumon should be stopped about 7 days or warfarin 5 days before the intervention. It should be checked on the day of the intervention that the INR is < 1.5 [4].

In patients who are anticoagulated with a DOAC, bridging with heparin is generally not necessary or useful. Studies have shown increased rates of bleeding complications with heparin bridging without any benefit in terms of the rate of thromboembolism [24, 25]. Of particular note is the PAUSE trial, which prospectively investigated a simple and standardized interruption of DOAC therapy in patients with atrial fibrillation without heparin bridging. It did not detect any relevant risk of bleeding or thrombosis [26].

For patients undergoing treatment with a VKA, this should be discontinued 5 days before the procedure and, if there is a high thromboembolic risk, bridging with LMWH is implemented. If there are no complications after the operation, therapy with the VKA can be continued after 3–4 days. DOACs have an advantage in that the planned intervention can be carried out during the dosing interval. If renal function is normal, a break of 24–48 h after the last dose (provided there are no drug interactions that prolong the half‐life) is usually sufficient to perform the procedure without an increased risk of bleeding. It is now also possible to measure the specific serum activity of each individual DOAC using a modified anti‐Xa test. There is early evidence that an apixaban level < 50 ng/mL could be considered safe for surgery [27]. Bridging is usually not necessary for patients who take DOACs. The RELY study reported that the thromboembolic risk for dabigatran was comparable with and without bridging, but bleeding occurred significantly more frequently in the bridging group [26].

We didn't examine the perioperative bridging of anticoagulation, as it based not only on periprocedural bleeding but also on thromboembolic risk [28]. Regarding the time point of VKA interruption, more than 70% of respondents interrupted VKAs at least 5 days before surgery. This is in accordance with the BRIDGE trial, which showed non‐inferiority of interrupted VKA compared to perioperative bridging with low‐molecular‐weight heparin for the prevention of arterial thromboembolism and simultaneous decreased risk of major bleeding [29].

## 5 Limitations

This study has some limitations. It is a questionnaire‐based work, which depends on the care of the respondents. The participants belonged to the different levels of hospital service, and we didn't evaluate if the reported strategy was the common rule in the hospital if arterial peripheral interventions has been conducted by other departments (like angiology or interventional radiology). The questionnaires have also not been evaluated. The response rate was 52%. Finally, the survey was performed in 2018, before COVID 19 Era, so nowadays some local strategies may have changed. Due to the large amount of data, we concentrated the questionnaire on timing of drug pauses before open surgery and omitted questions regarding percutaneous interventions. There are some unexplained discrepancies between sections in the answers concerning the pausing of medications.

## 6 Conclusions

Perioperative antithrombotic strategies in German vascular surgery clinics were not uniform to the timepoint of the survey in 2018 and didn't correspond to the specialist recommendations in a notable proportion of the departments/units.

## Author Contributions

**Dmitriy I. Dovzhanskiy:** conceptualization, writing – original draft, methodology, validation, visualization, formal analysis, project administration. **Moritz S. Bischoff:** writing – review and editing, supervision. **Karola Passek:** writing – original draft, validation. **Hinrich Böhner:** writing – review and editing, conceptualization. **Dittmar Böckler:** conceptualization, writing – review and editing, supervision.

## Conflicts of Interest

Authors state that there is no conflict of interest for any of the authors. All authors have read and approved the final version of the manuscript. Corresponding author had full access to all of the data in this study and takes complete responsibility for the integrity of the data and the accuracy of the data analysis.”

## Transparency Statement

The lead author Dmitriy I. Dovzhanskiy affirms that this manuscript is an honest, accurate, and transparent account of the study being reported; that no important aspects of the study have been omitted; and that any discrepancies from the study as planned (and, if relevant, registered) have been explained.

## Data Availability

The data that support the findings of this study are available from the corresponding author upon reasonable request.
